# *Parietaria* Allergy: An Intriguing Challenge for the Allergist

**DOI:** 10.3390/medicina54060106

**Published:** 2018-12-07

**Authors:** Giorgio Ciprandi, Paola Puccinelli, Cristoforo Incorvaia, Simonetta Masieri

**Affiliations:** 1 Ospedale Policlinico San Martino, 16132 Genoa, Italy; 2 Scientific, Pharmacovigilance and Regulatory Department Stallergenes-Greer, 20100 Milan, Italy; paola.puccinelli@stallergenesgreer.com; 3 Cardiac/Pulmonary rehabilitation Unit, ASST Pini/CTO, 20100 Milan, Italy; cristoforo.incorvaia@gmail.com; 4 Department of Otorhinolaryngology, Sapienza University, 00100 Rome, Italy; simonetta.masieri@uniroma.it

**Keywords:** *Parietaria*, pollen, allergy, symptoms, inflammation, allergen immunotherapy

## Abstract

*Parietaria* pollen is the most important cause of pollen allergies in the Mediterranean area, as *Parietaria* is widespread in this region. Many issues are associated with *Parietaria* allergy, including the duration of the pollen season (many doctors in fact believe that it lasts throughout the year), pollen load (which seems to be increasing over time), the impact of age (on IgE production and symptom severity), inflammatory changes (after pollen exposure), and the choice of allergen immunotherapy (AIT). In addition, molecular diagnostics allows for the defining of a correct diagnosis, differentiating between mere sensitization and true allergy. This review considers these topics and will hopefully help the allergist in clinical practice. *Parietaria* allergy is an intriguing challenge for the allergist in clinical practice, but it may be adequately managed by knowing the peculiarities of respective territories and the clinical characteristics of each patient.

## 1. Introduction

*Parietaria* allergy is a frequent manifestation of pollen allergy arising mainly in the Mediterranean area [1]. *Parietaria* allergy has some peculiar characteristics, including relevant epidemiological impact and long duration of symptoms (caused by the pollination period consistently being long-lasting). Asthma and rhinitis are frequently associated with the allergy, and have been treated with allergen immunotherapy for a long time [2,3]. With regard to these issues, it is important that this review present and discuss the most relevant clinical and therapeutic aspects.

## 2. *Parietaria* Pollen Impact on Allergy Patients

It is well known that the *Parietaria* plant is a weed that is widespread in the Mediterranean area, and that many people are allergic to it: it is responsible for up to 25–30% of all allergy patients [1]. *Parietaria* belongs to the Urticaceae family. Although many species of it exist, the most relevant are *P. officinalis* and *P. judaica*, if concerned with allergy pathogenesis. The term “parietaria” derives from the Latin word *paries*, meaning wall, as it easily grows in the shade of old walls. Another name for the plant is *Parietaria* “*vetriola*”, as it has historically been used for cleaning glass bottles.

The *Parietaria* pollen is small, and its mean diameter is measured in microns (more than PM_10_). As a consequence, only some grains can penetrate to the trachea, and none are able to reach the terminal bronchioles. Thus, a high frequency of asthma may be induced by bronchial inflammation deriving from inflammatory events occurring in the upper airways, as well as by other immunological mechanisms (such as, probably, paucimicronic particles carrying allergens). Indeed, patients allergic to *Parietaria* also frequently suffer from asthma [2]. However, a crucial issue which should be noted is that when atmospheric pollen grains are identified under an optical microscope, *Parietaria* pollen is not distinguishable from the pollen of most species of the genus *Urtica*. Consequently, these pollen grains may be identified as the pollen type Urticaceae. This point may suggest a need for caution in interpreting data reported in many studies, as overestimates of the number of *Parietaria* pollen grains may persist.

Furthermore, there is agreement regarding the concept that the prevalence of allergic disorders is growing. Because of this, many pathogenic theories have been hypothesized, including one positing an increase in pollen load. A five year aerobiological study, conducted from 1984 to 1988, indicated that *Parietaria* allergy is a noticeable issue in Italy, unlike in most other European countries, due to the duration and the quantity of pollen production [3]. More recently, a study demonstrated that the *Parietaria* season has tended to be prolonged, and its pollen count has tended to increase over time [4]. A second study took place over a 30-year period in the same geographic region [5]. Its main outcomes provided evidence that sensitization prevalence to *Parietaria* did not change as significantly as *Parietaria* pollen counts over time, but that the *Parietaria* pollen season was prolonged, a fact that is due to climatic and environmental change over the same period.

In this regard, the *Parietaria* pollination season may be rather long, and there is a popular belief that the symptoms for *Parietaria* allergy may be present for the whole year. This belief may have a practical implication for allergen immunotherapy (AIT) prescription. In fact, many doctors prefer to prescribe AIT for *Parietaria* allergy patients using a continuous schedule, while usually prescribing pre-co-seasonal AIT courses for allergies to other pollens. Though dependent on climatic factors, the actual duration of *Parietaria* pollen season is never perennial. Indeed, the *Parietaria* pollen season usually has two peaks: a main peak during spring and a second during early autumn. In autumn, there is a low peak in comparison to the very high concentrations observed in spring. A recent study addressed the assessment of the actual duration of the *Parietaria* pollen season in Italy, and was undertaken over a 10-year observation period [6]. Its findings show that *Parietaria* pollination lasts 6–7 months on average, with two main peaks seen: an important peak during mid-spring and a lower peak during early fall. However, there is remarkable variation within different geographic areas. These differences are obviously dependent on the climatic characteristics of each region. Interestingly, these outcomes are substantially consistent with those found in previous surveys. 

The seasonality of *Parietaria* pollen was confirmed by a study that demonstrated a clear association between pollen exposure and nasal inflammation in patients allergic to *Parietaria* [7]. This phenomenon can be easily interpreted by considering the idea that allergic inflammation is closely dependent on pollen exposure [8]. In fact, the duration of nasal allergic inflammation typically mirrors the duration of the pollen season for patients with *Parietaria* allergy. 

This concept may have practical consequences for approaches to the treatment of patients with *Parietaria* allergy, mainly with regard to the AIT schedule. In relation to this, two studies have recently been published. The first study demonstrated that a cycle schedule was as effective as a continuous course [9]. The second confirmed that a single pre-co-seasonal sublingual immunotherapy (SLIT) *Parietaria* course could be sufficient to reduce symptom severity and medication use [10]. 

Another interesting issue is the impact of age on allergen-specific IgE production. Some studies conducted in Italy have confirmed that sensitization to *Parietaria* pollen does not significantly decrease with age [11,12]. In other words, IgE production due to *Parietaria* pollen persists until late age. This fact has a clinical consequence: it has been reported that the severity of allergic symptoms is slightly affected by age for *Parietaria* allergy patients, whereas patients with house dust mite allergies frequently perceive less severe symptoms after 40 years of age, as mite sensitization significantly diminishes after this age [13]. 

Molecular diagnostics is a crucial step in the work-up of allergy patients, mainly with regard to AIT prescription [14]. Defining the molecular pattern of sensitization allows for discrimination between genuine sensitization and that which is spurious, thus avoiding ineffective AIT prescriptions. Additionally, serum IgE level assessment may be useful for differentiating true allergies from mere sensitization [15]. There is evidence that measurement of the level of IgE to Par j 2 allows for the recognition of allergic subjects [16].

## 3. Allergen Immunotherapy for *Parietaria* Allergy

The first controlled trial on the efficacy and safety of subcutaneous immunotherapy (SCIT) dates back to 1994. In this trial, 18 patients with rhinoconjunctivitis caused by *Parietaria* pollen were treated with a partially purified alginate-conjugated extract of *P. judaica,* or a placebo. During the *Parietaria* pollen season, actively treated patients had significantly lower nasal symptom/medication scores and significant decreases in specific, nasal and conjunctival reactivity to the *Parietaria* extract [17]. A year later, the first double-blind trial on the effects of SLIT on *Parietaria*-induced rhinitis was published; it included 31 patients randomized to active or placebo treatment. When comparing data obtained before and after a 10-month period of treatment, it can be observed that actively treated patients had significantly lower medication scores during maximum *Parietaria* pollen counts, as well as a significant decrease in nasal reactivity by the end of the study [18]. Indeed, these controlled studies were preceded by a trial on 38 patients allergic to *Parietaria* who were treated with local nasal immunotherapy (LNIT) or a placebo. Positive outcomes were reported for medication scores (but not symptom scores) and nasal reactivity to *Parietaria* [19]. However, this route of administration is no longer used because of the high rate of local nasal reactions to treatment. As for SCIT and SLIT, several additional controlled studies were performed in the following years. Table 1 and Table 2 summarize the main observations from trials on SCIT [20,21,22,23,24,25,26] and on SLIT [27,28,29,30,31,32,33]. Similarly to what has been done for the most important allergens in recent years, the search for better quality immunotherapy products has resulted in a series of scientific publications, the results of which are summarized here. One important aspect regards the safety and tolerability of the two routes of administration. For SLIT the available data indicate a greater frequency of local reactions in the oral cavity, but not of systemic reactions [34]. Regarding SCIT, a prospective 3-year study on 510 patients treated with mono-phosphoril lipid-associated allergoids as adjuvant reported a frequency of systemic reactions (none of them serious) of 1.37% [35]. However, severe reactions cannot be ruled out, especially if ultra-rush patterns are used [36]. Approaches proposed in recent years to improve both the efficacy and safety of *Parietaria* pollen immunotherapy consist of using innovative materials but also the development of techniques able to increase diagnostic precision and therefore to improve prescription appropriateness. With regard to the first approach, hybridization techniques employing genetic engineering using fragments of the two major allergens of *P. judaica* (Par j 1 and Par j 2) allowed for the obtaining of proteins with reduced allergenic activity, which were associated with reduced skin reactivity [37]. In an Italian experimental study, recombinant molecules with variants of Par j 1 and Par j 2, obtained by genetic engineering, proved to be markedly less reactive while retaining the ability to stimulate T cells and the production of IgG antibodies in a similar way to the natural extract of pollen [38]. Another promising technology may be found in nanoparticles, which can be used as protein vehicles. Using solid lipid nanoparticles, the recombinant molecule of Par j 2 has demonstrated high safety in vitro [39]. The same group of researchers also studied a nano-aggregated allergenic copolymer, which likewise demonstrated safe application in human tissues, in addition to an ability not to alter the capacity to cross-link with specific IgE and therefore not to interfere with the activity of allergen-induced biology [40]. Finally, a further form of hybridization of Par j 1, Par j 2, and their isoforms has shown that the recombinant hetero-dimeric Par j heterodimer (PjED), which may be obtained from the shorter isoform of Par j 1 allergen, induces the synthesis of IgG1. IgG1 is able to bind to all major allergic isoforms of *Parietaria*, representing a promising candidate for immunotherapy [41]. It must be stated that all these forms of immunotherapy have yet to reach the development phase for possible use in clinical practice.

The other important field of application is represented by the advanced techniques available for precision diagnosis. In 2007, the group Valenta suggested that molecular diagnostics (also referred to as component-resolved diagnosis (CRD), or simply molecular diagnostics, as previously reported) can be particularly useful with regard to pollinosis from plants such as *Parietaria*, which often affects polysensitized patients. With these patients, it is essential to identify the specific molecules of the respective pollen, distinguishing them from molecules of other pollens and facilitating a more appropriate prescription of AIT [42]. It has been reported that the presence in subjects with positive allergy tests of specific IgE to Par j 2 makes it possible to distinguish clinical allergies from simple sensitization [43]. This is particularly relevant from a clinical point of view, as it allows for the accurate obtaining of an appropriate AIT prescription. Another molecular allergy study on individuals with positive allergy testing for *Parietaria* has shown that the specific culpability of *Urtica dioica* (an Urticaceae species which is cross-reactive with *Parietaria*), which contains species-specific allergens such as osmotin (a taumatin-like protein) and pectin-esterase, can be assessed by CRD, thus avoiding the prescription of immunotherapy with a low probability of success [44]. Finally, one study has demonstrated the usefulness of the basophil activation test (BAT) in patients sensitized to *Parietaria*. In particular, among the different activation markers, CD203c appeared to be related to the presence of symptoms, allowing for the identification of in vitro patients with asymptomatic sensitization [45]. 

For patients treated with immunotherapy, serum levels of interleukin 9 (IL-9) have proven useful for indicating treatment efficacy. In fact, when comparing patients allergic to *Parietaria* who were treated with SLIT to those treated with drugs, significantly lower levels were found in patients treated with SLIT [46]. This is particularly important with regard to clinical practice because there is a need to measure biomarkers which are potentially able to a priori select responders to AIT and to evaluate AIT effectiveness [47,48]. 

Studies defined as “real-life”, due to their employment of common clinical practice patients and not patients chosen via the rigid and selective methods of double-blind placebo-controlled trials, provide data of great value to physicians interested in AIT. In one study of patients with mild asthma, a cycle of SCIT based on four injections of *Parietaria* extract with mono-phosphoryl lipid as adjuvant obtained an improvement in asthma control, and was measured using the questionnaire “Asthma control test” [49]. In another real-life study of high dose SLIT with standardized extracts (Staloral 300IR) in patients with various types of pollinosis, *P. judaica* was the most widely used, along with grasses and Cupressaceae. Patients suffered from severe allergic rhinitis (in 55% of cases there was mild asthma) and were evaluated after 12 and 36 months of treatment by considering their total score of nasal symptoms and drug use, and, in patients with asthma, asthma symptoms and consumption of asthma drugs. There were statistically significant differences from baseline values for rhinitis symptoms (*p* < 0.041), consumption of drugs for rhinitis (*p* < 0.0162), asthmatic symptoms (*p* < 0.0162), and consumption of asthma drugs (*p* < 0.0005) [50]. 

Studies have also been undertaken on two important aspects of immunotherapy: effects on quality of life, and pharmaco-economic aspects. One study prospectively evaluated 167 polysensitized patients, in which the most common causes of sensitization were grass pollen, *Parietaria* pollen and dust mites. Quality of life was measured using the “Rhinoconjunctivitis quality of life” questionnaire, which was taken once before starting and once after a year of treatment. SLIT was performed using a single extract for 123 patients (73.6%), two extracts for 31 patients (18.6%) and more than two extracts for only 13 patients (7.8%). Quality of life improved significantly (*p* < 0.01 compared to the baseline) in all cases [51]. Another pharmaco-economic study examined 30 patients who suffered from rhinitis and asthma due to *Parietaria*, 20 of whom were treated with SCIT with an extract of *Parietaria judaica* (Alustal, Stallergénes) and 10 of whom were treated with antiallergic drugs. All patients were evaluated before starting and then annually for a duration of six years, based on allergic symptoms during the *Parietaria* pollination period and the consumption of drugs. A significant difference was observed in favor of immunotherapy, with a reduction in treatment costs of approximately 15% in the second year and 48% in the third year. Three years after SCIT was stopped the reduction in costs reached 80%, with a net saving for each patient that corresponded to €623 [52].

Another interesting aspect is the tolerability of SCIT: a very recent study demonstrated that *Parietaria judaica* subcutaneous immunotherapy (Allergovac^®^ depot ROXALL Medicina España), with an abbreviated up-dosing scheme, showed an adequate safety and tolerability profile and induced preliminary efficacy changes [53]. This indicates that a rapid schedule may be safe and well tolerated.

Finally, a new therapeutic strategy approach may be the design of standardized recombinant hypoallergenic derivatives which can reduce allergic adverse events. A recent study using mice demonstrated the capability of an engineered hybrid, based on the disruption of disulphide bonds in the allergen molecule, to modulate pre-existing sensitization towards a protective immune response [54]. These findings could represent a new model applicable to other allergen molecules belonging to the lipid transfer protein family.

## 4. Conclusive Remarks

*Parietaria* allergy is very common in the Mediterranean area, especially in Italy. It has specific peculiarities, mainly concerning the duration of its pollen season and the persistence of symptoms in elderly people. AIT is usually effective and new potential treatment schedules could possibly be performed. However, as AIT is a perfect example of Personalized Medicine, it is fundamental to use a pragmatic approach based on Precision Medicine in order to define specific biomarkers of clinical utility for the management of AIT, as has been recently pointed out [55]. 

Moreover, there is a body of evidence indicating that many factors are involved in the increased propensity of people to overreact to previously innocuous and tolerated antigens. These factors include environmental influence, developmental plasticity origins, and the hygiene hypothesis. It is well known that pollution is a significant factor that can modify climate characteristics, which in turn may significantly affect the vegetable kingdom, and critically, *Parietaria* pollen production [56,57]. The environment can also play a role as phenotype inducer so that a single genotype can become able to express an alternative appropriate phenotype, as a consequence of environmental variation [58]. This phenomenon is defined as developmental plasticity, which is often heritable, and may evolve under selective mechanisms [59]. Hence, people can produce integrated, adaptive, and environment-specific phenotypes as natural evolution resulting from changes in global climate occurs [60]. In this regard, the hygiene hypothesis has produced a unanimous body of evidence which explains the allergy epidemics. This theory was proposed initially by Strachan who speculated that exposure to frequent infections in large families could be a protective factor for allergy onset [61]. The hygiene hypothesis has evolved toward a more contemporary “biodiversity hypothesis” that looks beyond the effect of both infections and single microbes to the potential protective effect of gut colonization with diverse environmental microflora; the term “microbiota hypothesis” has been proposed [62]. Overall, environmental factors, developmental plasticity, and the microbiota hypothesis may have modified the immune response to allergens from a tolerance condition to IgE-mediated hypersensitivity.

In conclusion, *Parietaria* allergy is an intriguing challenge for the allergist in clinical practice, but it may be adequately managed by knowing the peculiarities of the respective territories and the clinical characteristics of each patient.

## Figures and Tables

**Table 1:** Controlled trials on subcutaneous immunotherapy with *Parietaria* extracts.

Author, Year [Ref]	Study Population	Issue Addressed	Results
D’Amato et al., 1995 [20]	20 adult pts	Efficacy and safety	Significant decrease of nasal symptoms, comparable incidence of systemic reactions in active and placebo treatment
Tari et al., 1997 [21]	40 adult pts	Efficacy and safety	Improvement in nasal inspiratory peak flow, low incidence of side effects
Ariano et al., 1999 [26]	25 patients of all ages	Efficacy and safety	Significant reduction of symptom-medication scores (SMS), decrease of sIgE and increase of sIgG4; no important side effects
Garcia-Selles et al., 2003 [22]	30 adult pts	Efficacy and safety	Significant difference in SMS in actively vs. placebo treated; no side effects.
Crimi et al., 2004 [23]	30 adult pts	Efficacy and effect on bronchial hyper-responsiveness (BHR)	Significant difference in SMS in actively vs. placebo treated; no change in BHR
Polosa et al., 2004 [24]	30 adult pts	Efficacy, effect on BHR and sputum eosinophils.	Significant difference in SMS in actively vs. placebo treated; no change in BHR and sputum eosinophils.
Ferrer et al., 2005 [25]	42 adult pts	Efficacy and safety	Sustained decrease in SMS in actively treated; no side effects.

**Table 2:** Controlled trials on sublingual immunotherapy (SLIT) with *Parietaria* extracts.

Author, Year [Ref]	Study Population	Issue Addressed	Results
Purello D’Ambrosio et al., 1996 [27]	40 adult pts	Efficacy and safety	Significantly lower SMS in SLIT treated; no side effects
Passalacqua et al., 1999 [28]	30 adult pts	Efficacy and safety	Significant reduction of symptom score and drug intake in the active group; no side effects
Purello D’Ambrosio et al., 1999 [29]	30 adult pts	Efficacy and safety	Significantly lower symptom and drug scores in SLIT treatment; no side effects
La Rosa et al., 1999 [30]	41 children	Efficacy and safety	Significant reduction in symptom but not in medications scores; increase in the threshold dose for conjunctival allergen provocation test; no side effects
Pajno et al., 2003 [31]	38 children	Comparison of efficacy of SLIT and fluticasone in *Parietaria* induced asthma	Equal efficacy of SLIT and fluticasone, only SLIT efficacious also in rhinitis
Pajno et al., 2004 [32]	30 children	Effect on seasonal BHR.	Abrogation of seasonal BHR in actively treated children
D’Anneo et al., 2008 [33]	65 adult pts	Efficacy and effect on BHR of two SLIT dosages compared to drug therapy; safety	Significant improvement in symptoms, drug use and BHR in both SLIT group; no side effects

SMS: Symptom mean score; BHR: Bronchial Hyperreactivity.
